# *In Vivo* Therapeutic Potential of Next-Generation Probiotic *Akkermansia muciniphila* and Butyrate Combination Therapy in Diabetes

**DOI:** 10.4014/jmb.2506.06025

**Published:** 2025-10-28

**Authors:** Fatemeh Ghorbanian, Hoonhee Seo, Faezeh Sarafraz, Ali Atashi, Md Sarower Hossen Shuvo, Park Chae-eun, Mohammed Solayman Hossain, Hanieh Tajdozian, Sukyung Kim, Ho-Yeon Song, Heejo Yang

**Affiliations:** 1 Department of Microbiology and Immunology, School of Medicine, Soonchunhyang University, Cheonan-si, Chungnam 31151, Republic of Korea; 2 Human Microbiome Medical Research Center (HMMRC), School of Medicine, Soonchunhyang University, Asan, Chungnam 31538, Republic of Korea; 3 Department of Urology, School of Medicine, Soonchunhyang University, Cheonan-si, Chungnam 31151, Republic of Korea

**Keywords:** Type 1 diabetes mellitus (T1DM), *Akkermansia muciniphila*, butyric acid, next-generation probiotic, SD rat, C57BL/6J mice

## Abstract

Recent studies have found that gut microbiota is closely related to the initiation and progression of T1DM. This study aimed to examine the effects of sodium butyrate and *Akkermansia muciniphila* (AKK), both individually and combined, on T1DM models. To optimize a T1DM model, a single high dose of streptozotocin (STZ) was administered to C57BL/6J mice to determine the appropriate dose (150 mg/kg). Mice were divided into four groups: a control group, a diabetic group, a diabetic group treated with butyrate, and a diabetic group treated with AKK. The groups treated with butyrate and AKK reduced blood glucose levels, prevented body weight loss, and normalized food and water intake. To explore the synergistic effects of AKK and butyric acid, Sprague-Dawley (SD) rats were assigned to control, diabetic, and treatment groups including butyrate, AKK, and combination therapy. Two weeks of oral treatment showed improved metabolic parameters in the treatment groups. The combination therapy exhibited the most improvements. In the *in vitro* experiment, the cytotoxic effects of STZ on 3T3-L1 cells treated with both AKK and butyric acid were measured, indicating their protective effects on cell viability. GC–MS/MS analysis was also performed to assess changes and correlations in systemic short-chain fatty acids (SCFAs). SCFAs were significantly increased in the liver after probiotic treatment, especially in the combination therapy group. These findings demonstrate the microbiota-regulating and therapeutic effects of AKK and butyrate, particularly highlighting the potential of combination therapy as an alternative to standard diabetic treatments.

## Introduction

Type 1 diabetes mellitus (T1DM), previously known as juvenile or insulin-dependent diabetes, is a chronic autoimmune disease resulting from the destruction of pancreatic β-cells, leading to insufficient insulin and hyperglycemia [1, 2]. Effective diabetes management is crucial. Traditional treatment aims to reduce blood glucose and relieve symptoms to enhance quality of life, including insulin, insulin analogs, peptides, and oral hypoglycemic therapies [3]. However, the side effects of these therapies, along with challenges in accessibility and affordability, particularly in low- and middle-income countries, emphasize the need for alternative approaches [3].

The human gut is a complex ecosystem composed of nutrients and inhabited by approximately 100 trillion bacteria known as the gut microbiota [4]. Over the last few years, the gut microbiomés functions in health and disease have been the subject of much research [4, 5]. According to FAO/WHO, “probiotics are live bacteria or microorganisms that, when administered in sufficient amounts, can support and improve host health.” [6].

Among different probiotics, *Akkermansia muciniphila* (AKK), a Gram-negative, strict anaerobe that constitutes 0.5–5% of the total gut microbiota, has attracted interest as a next-generation probiotic due to its ability to influence health and its key role in preventing and alleviating conditions such as obesity, diabetes, metabolic syndrome, inflammation, and even aging-related disorders [7-9].

Moreover, gut bacteria can synthesize short-chain fatty acids (SCFAs), including butyric acid and propionic acid, which play key roles in gut and immune health [10]. Butyrate helps maintain intestinal barrier integrity, reduces local inflammation, and serves as an energy source for colonic epithelial cells. A lack of butyrate is also linked to several intestinal disorders and immune dysregulation [10-12]. Individual effects of AKK and butyrate have been extensively studied; however, their combined therapeutic potential in T1DM remains largely unexplored, with limited in *vivo* evidence demonstrating their synergistic effects. Streptozotocin (STZ) is commonly used to induce cytotoxicity and mimic conditions of T1DM by damaging cells and disrupting metabolic pathways. This study aimed to evaluate the therapeutic potential of AKK and sodium butyrate, individually and in combination, in STZ-induced T1DM mouse and rat models.

## Materials and Methods

### Bacterial Strain and Optimization of Growth Conditions

The AKK strain KCTC 15667 was obtained from the Korean Collection for Type Cultures (KCTC), South Korea. Bacterial cells were received and cultured in Brain Heart Infusion medium (BD, USA) supplemented with 0.4% porcine gastric mucin (Sigma-Aldrich, China) [13]. AKK was incubated at 37°C under four different atmospheric conditions to evaluate the growth pattern. AKK cultures were grown in duplicate under each condition.

A strict anaerobic environment was maintained using a Concept 400 anaerobic workstation (Baker Ruskin, Canada). Additionally, an anaerobic jar system (Kisan Bio, Republic of Korea) with commercial anaerobic gas-generating bags was used to create an oxygen-free atmosphere. Microaerophilic conditions were achieved with a Concept 450 microaerophilic chamber (Baker Ruskin), which provided limited oxygen exposure. As a control, cultures were incubated under standard aerobic conditions in the N-Biotek incubator at 37°C. Colony-forming units (CFU) per milliliter were counted at 6-hour intervals from 12 to 60 h by serial dilution and plating on BHI-mucin agar, followed by anaerobic incubation for 48–72 h. Colonies were tallied and recorded in standard number format; a growth curve was constructed to determine the log of the growth phase.

### Animal Selection and Experimental Design

Male C57BL/6J mice (5 weeks old) and Sprague-Dawley (SD) rats (7 weeks old, weighing 190-220 g) were purchased from Doo Yeol Biotech Company, Korea. These animals were maintained under standard laboratory conditions for the entire study period. The light-dark cycle in the animal facility comprised a 12-h light: 12-h dark cycle. The temperature was 22 ± 2°C, and the humidity was 45-55%. The animals were given free access to tap water and a regular diet for one week of adaptation before the start of the experiment. To evaluate how diabetes developed without treatment and fine-tune the experimental setup, we first conducted a small optimization study using STZ (Med Chem Express, USA) via an injection method in mice [14]. Following successful optimization in mice, we began the treatment phase in mice. After that, we initiated the rat treatment protocol to check the efficacy of probiotics, butyric acid, and their combination therapy.

### Establishment of Diabetes Using STZ in Mice

Mice were divided into control and diabetic groups receiving different STZ doses (100 mg/kg, 150 mg/kg, 200 mg/kg) (*n* = 5 mice per group). All mice, aged 6 weeks, were fasted for 4 h before injection. Control mice received a single intraperitoneal (IP) injection of citrate buffer (pH 4.5) (Sigma-Aldrich, Germany), while the diabetic groups received a single IP injection of STZ at their respective doses. Blood glucose levels were measured using the Accu-Chek active glucometer (Roche Diagnostics Care GmbH, Germany) before STZ injection, and monitored every 3 days thereafter using tail vein blood samples, following a 4-h fasting period. Body weight and illness severity were checked every three days, and food consumption and water intake were monitored weekly.

### Evaluation of AKK and Butyrate Treatment in Diabetic Mice

Male mice were randomly divided into four groups: control, T1DM, probiotic treatment, and sodium butyrate treatment (*n* = 5 mice per group). All mice were fasted for 4 h before injection. The T1DM and treatment groups received a single IP injection of STZ (150 mg/kg), while the control group received an equal volume of sodium citrate buffer (pH 4.5) (Sigma-Aldrich, China). One week after the STZ injection, treatment was initiated. The probiotic group received AKK at 1 × 10^9^ CFU/200 μl/mouse, and the sodium butyrate group received sodium butyrate (500 mg/kg) (Sigma-Aldrich) once daily. Blood glucose was measured from tail vein samples using a glucose meter. Mice with glucose levels > 250 mg/dL were classified as diabetic. Glucose levels were monitored every 3 days, and food and water intakes were monitored weekly for the duration of the experiment.

### Investigation of Probiotic, Butyric Acid, and Combination Therapy in a Diabetic Rat Model

Rats were randomly divided into control, T1DM, probiotic, sodium butyrate, and combined treatment groups (*n* = 5 rats per group). After 8 h of fasting, diabetes was induced by a single IP injection of STZ (65 mg/kg) in all groups except controls, which received sodium citrate buffer. This dose was chosen based on previous studies to ensure high efficacy and survival rates [15].

Fasting blood glucose was measured 3 days after injection; rats with fasting serum glucose levels above 250 mg/dl were considered diabetic and used in the subsequent experimental procedures [16]. Rats in the probiotic group received AKK at 1 × 10^9^ CFU in 1 ml per rat. Rats in the sodium butyrate group received sodium butyrate at 500 mg/kg. Rats in the combined group received the AKK and sodium butyrate at the same doses as the probiotic and sodium butyrate treatment groups once daily for 2 weeks [17, 18]. Clinical signs, fasting blood glucose levels, body weight, and food and water intake were monitored weekly.

### Review of Animal Experiment Ethics

All animal experimental procedures were conducted in a Biosafety Level 2 facility (LML 20–591) at Soonchunhyang University per the guidelines of the Ministry of Food and Drug Safety (MFDS Registration No. 657). The Institutional Animal Care and Use Committee (IACUC) of Soonchunhyang University reviewed and approved experimental protocols for using animals. Separate approvals were obtained for mice (SCH24-0050) and rats (SCH24-0069), depending on the study design.

### Liver SCFAs and Metabolite Profiling Analysis

SCFAs, including propionic acid, butyric acid, acetic acid, and valeric acid in liver samples, were analyzed using gas chromatography‒mass spectrometry/mass spectrometry (GC–MS/MS) (GC: 2010 Plus, GC-MS: TQ8040; SHIMADZU, Japan). Briefly, liver samples (20 mg) were mixed with 30 μl of 0.1 M NaOH and 430 μl of methanol in an eppendorf tube. The mixture was incubated at -20°C for 20 min. After incubation, the sample was centrifuged at 22,000 ×*g* for 10 minutes at 4°C, and 450 μl of the supernatant was collected. The collected solution was completely dried at 37°C. MeOX was dissolved in pyridine to a final concentration of 20 mg/ml, and 40 μl was added to the dried sample. The reaction was allowed to proceed at 60°C for 90 min. Subsequently, 60 μl of the derivatization reagent MTBSTFA was added, and the sample was incubated at 60°C for 30 min. The final test solution was centrifuged at 22,000 ×*g* for 10 min at 4°C. Then, 70 μl of the supernatant was mixed with 140 μL of pyridine [19, 20]. The final solution was subjected to GC-MS/MS analysis.

### Examining the Efficacy of a Candidate Probiotic Strain to Overcome STZ Effects

The 3T3-L1 cell line, a mouse fibroblast cell line, was purchased from the Korean Cell Line Bank. Cells were cultured in Dulbeccós Modified Eagle Medium (DMEM, Gibco, USA) supplemented with 10% fetal bovine serum (FBS, Gibco, USA) and incubated at 37°C in a 5% CO_2_ atmosphere for 12 h. Following this incubation, cells were treated with STZ (5 mM, freshly prepared in citrate buffer, pH 4.5) for 24 h before being subjected to different experimental conditions. For confocal microscopy analysis, cells were seeded into 96-well tissue culture plates. After 24 h of incubation, cells were washed with phosphate-buffered saline (PBS) and cultured under various treatments, including AKK (10^7^ CFU/ml) and sodium butyrate (5 mM). Following overnight incubation, cells were stained with propidium iodide (PI) solution (50 μg/ml) for 15 min, counterstained with DAPI, and visualized under a fluorescence microscope (Olympus) to capture images [21].

### Cell Viability Assay

To assess cell viability, 3T3-L1 cells were cultured in 25 ml flasks containing DMEM supplemented with 10%FBS at 37°C and 5% CO_2_ for 12 h, as described previously. After reaching 70–80% confluency, cells were exposed to STZ (5 mM) for 24 h, followed by treatment with AKK (10^7^ CFU/ml) and sodium butyrate (5 mM). After 24 h of incubation, cells were washed with PBS, and cell viability was checked after trypan blue staining using a light microscope [21].

## Results

### Growth Pattern of AKK under Different Atmospheric Conditions

The growth of AKK was evaluated under four different environments: an anaerobic chamber, an anaerobic jar, a microaerophilic chamber, and aerobic conditions (Fig. S1). In the strictly anaerobic conditions (Fig. S1A), the strain grew strongly, with approximately 1.5 × 10^8^ CFU/ml at 36–42 h. The growth entered the stationary phase after 42 h, and then the count at 54 h slightly dropped. In the anaerobic jar setup (Fig. S1B), the growth profile reached maximum density (~7.6 × 10^7^ CFU/ml). The logarithmic phase started around 18 h and reached a peak at 36–42 h. Under microaerophilic conditions (Fig. S1C), growth was limited due to reduced oxygen availability, with a maximum of 6.8 × 10^7^ CFU/ml in 36 h. However, the CFU decreased after this point, suggesting that the entry into the stationary or dead phase occurred earlier compared to anaerobic conditions. Aerobic conditions did not result in the growth of the bacteria Fig. S1D. The CFU remained below the detectable level throughout the incubation period, indicating that AKK is strictly anaerobic and unable to grow in the presence of oxygen. Overall, the findings show that AKK requires strict anaerobic conditions for maximum yield, and in 36-42 h, it reaches the Logarithmic phase.

### Optimization of STZ Dose in Mice for Diabetes Induction and Their Effect on Physical Index

**Fig. 1:**
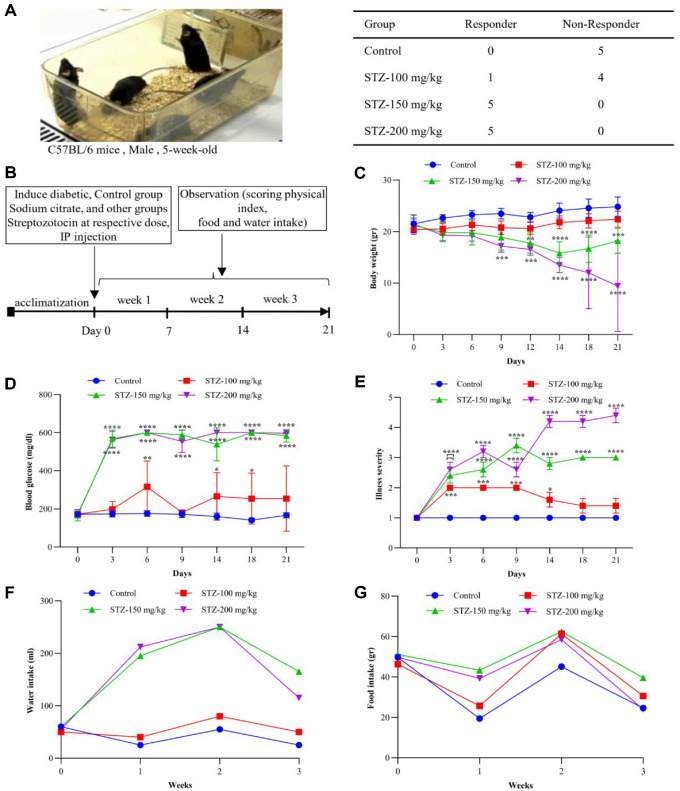
Dose-Dependent Effects of STZ in Mice. (**A**) Five-week-old C57BL/6 male mice were divided into control and STZ-treated groups (100,150,200 mg/kg). The table shows the experimental group assignment and the number of mice diagnosed with diabetes (responders) and those not diagnosed (non-responders) in each group. (**B**) After a one-week acclimation, mice in the STZ groups received a single intraperitoneal injection of STZ at the indicated dose (100, 150, or 200 mg/kg). In contrast, the control group received sodium citrate buffer (pH 4.0). Mice were monitored for three weeks postinjection. (**C**) Body weight was monitored during the study period. A significant reduction in body weight was observed in the 150 and 200 mg/kg STZ groups starting from the second week (***p < 0.001; ****p < 0.0001). (**D**) Blood glucose levels increased significantly three days post-injection in the 150 and 200 mg/kg groups compared to the control groups (****p < 0.0001). (**E**) Illness severity was assessed by monitoring mice in each group according to the following scoring system: (1 – healthy; 2 - minimally ill; 3 - moderately ill; 4 - severely ill; and 5 - dead). Mice treated with 200 mg/kg STZ showed two fatalities and higher illness scores. Mice in the 150 mg/kg STZ group exhibited diabetic conditions with higher severity but no mortality(****p < 0.0001). (**G**) Food consumption fluctuated throughout the study. Mice in the 150 mg/kg STZ group consumed more food compared to the other groups during the final week. (**F**) Water intake increased in the 150 and 200 mg/kg groups, corresponding to elevated blood glucose levels. Data are presented as mean ± SD (*n* = 5 mice per group). Statistical significance was evaluated by comparison with the control group, using two-way ANOVA with Tukey’s post hoc test (*p < 0.05; ***p < 0.001; ****p < 0.0001).

To determine the optimal dose of STZ and its effect on body weight, blood glucose levels, and signs of illness, mice were administered 100, 150, or 200 mg/kg STZ (Fig. 1). Fig. 1A shows the C57BL/6 mice in their cage. It includes a table presenting the number of mice identified as diabetic and non-diabetic. The 100 mg/kg STZ dose was insufficient to induce diabetes, as only 1 out of 5 mice responded as diabetic. In the 150 mg/kg group, all animals were found to be diabetic. At 200 mg/kg, all animals showed severe hyperglycemia with signs of toxicity in some mice. Fig. 1B shows the design of the experimental model.

All statistical analyses were performed using two-way ANOVA with Tukey’s post hoc test and compared with the control group. Data are presented as mean ± SD (Standard Deviation) (*n* = 5 mice per group). Throughout the test period, mice in the 100 mg/kg group maintained their body weights similar to those of the control group, while those in the 150 mg/kg and 200 mg/kg groups showed a gradual weight loss. On day 9, both the 150 mg/kg (*p* = 0.023) and 200 mg/kg groups (*p* = 0.0007) began to display weight reduction. On day 14, the weight loss in both groups became more significant. The 200 mg/kg group showed a greater degree of reduction in body weight (*p* < 0.0001) than the 150 mg/kg group (*p* = 0.0003) (Fig. 1C).

Blood glucose levels were measured throughout this study. Mice in the 100 mg/kg group had blood glucose levels close to those in the control group, while mice in the 150 mg/kg group showed higher levels indicative of diabetes. The 200 mg/kg group showed the highest blood glucose levels, which were associated with more severe diabetic symptoms, compared to all other groups starting at day 6 (*p* < 0.0001) and continuing through day 21 (*p* < 0.0001) (Fig. 1D).

Illness severity was evaluated by monitoring each group’s overall health and survival rate (Fig. 1E). Mice treated with 100 mg/kg STZ showed minimal illness and remained similar to mice in the control group (*p* = 0.0001). In contrast, the 200 mg/kg group exhibited a more severe diabetic phenotype (*p* < 0.0001), with two mice showing fatalities during the study. The 150 mg/kg group developed diabetic conditions with similar symptoms to those of the 200 mg/kg group but without fatalities (*p* < 0.0001).

Food and water intake were also monitored throughout the study period (Fig. 1F and 1G). Mice in both the 150 mg/kg and 200 mg/kg groups showed increased food and water consumption, consistent with their diabetic symptoms. In contrast, the 100 mg/kg group demonstrated no significant change in food or water intake compared to control mice. Based on these findings, a single dose of 150 mg/kg was chosen as a suitable dose to induce diabetes in male C57BL/6 mice.

### Effect of Probiotic and Butyric Acid Treatment on Physiological Parameters of Diabetic Mice

**Fig. 2:**
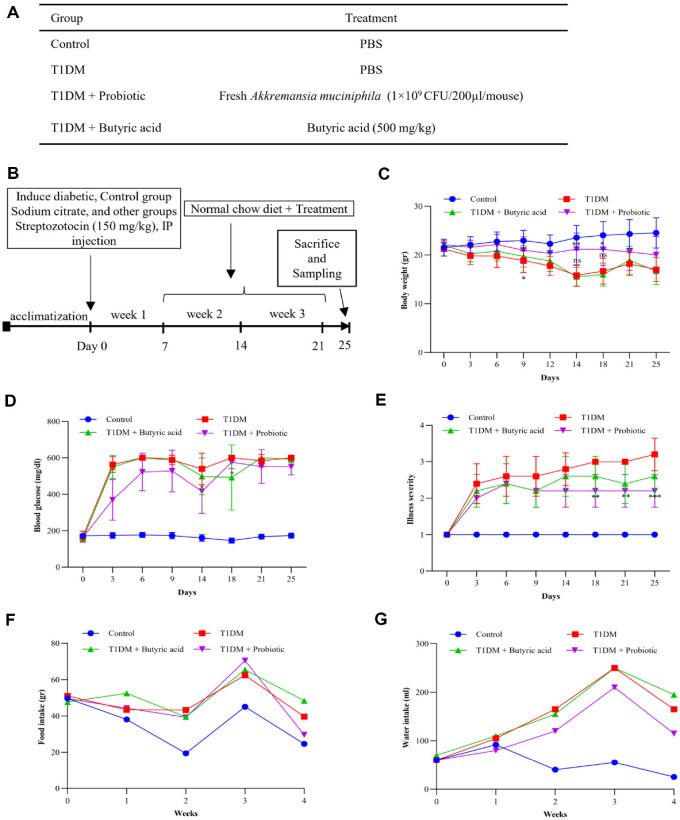
Effects of AKK and Butyric Acid on Physiological Index in a Type 1 Diabetes Mouse Model. (**A**) An overview of experimental groups is shown in the table. (**B**) Mice in T1DM and treatment groups received a single intraperitoneal injection of STZ (150 mg/kg) on day 0, control mice (diabetic mice without treatment) received sodium citrate buffer (pH 4.0), and treatment began on day 7 after diabetes confirmation. (**C**) Body weight was monitored throughout the study. Mice treated with AKK showed significantly improved weight maintenance compared to T1DM and butyric acid-treated groups. (**D**) Fasting blood glucose levels increased sharply in all STZ-injected groups by day 7. The AKK group’s glucose levels gradually decreased until day 14 of treatment and fluctuated afterwards (*p < 0.05). In contrast, the butyric acid and T1DM groups maintained elevated glucose levels. (**E**) The severity score for illness was evaluated using the following scoring system: 1 – healthy; 2 - minimally ill; 3 - moderately ill; 4 - severely ill; and 5 - dead. AKK-treated mice showed more significant positive scores than the butyric acid group (***p < 0.001). (**F, G**) Food and water consumption levels were monitored for the entire study. The T1DM group exhibited reduced food intake and increased water intake, while both treatment groups showed partial recovery, with AKK-treated mice displaying normalized levels. Data are presented as mean ± SD (*n* = 5 mice per group). Statistical significance was calculated using two-way ANOVA with Tukey’s post hoc test (*p < 0.05; **p < 0.01; ***p < 0.001; ****p < 0.0001 vs T1DM).

In this study, we investigated the therapeutic effects of AKK and butyric acid on T1DM and the physiological parameters of diabetic mice to assess the effects of experimental treatments over 25 days. Results of blood glucose level, body weight, illness severity, and food and water intake are shown in Fig. 2. Male C57BL/6 mice (5 weeks old) were randomly divided into four groups: control, T1DM, T1DM + probiotic, and T1DM + butyric acid (*n* = 5 mice per group) (Fig. 2A). After one week of acclimatization, STZ was injected intraperitoneally at a dose of 150 mg/kg to both the T1DM and treatment groups to induce diabetes. In contrast, sodium citrate buffer was injected into the control group of mice. Diabetic mice were defined as those with glucose levels higher than 250 mg/dL from day 7. Thereafter, daily oral treatment began by feeding the probiotic group with AKK at 1 × 10^9^ CFU/200 μl and the butyric acid group with sodium butyrate at 500 mg/kg (Fig. 2B). Data were analyzed using two-way ANOVA with Tukey’s post hoc test and compared with the T1DM group. Data are presented as mean ± SD (Standard Deviation).

Body weight showed a significant reduction starting from day 9 in the untreated T1DM group (*p* = 0.04), and continued to decline (*p* < 0.0001) (Fig. 2C). Treatment with butyric acid failed to show significant improvement in body weight compared to the untreated T1DM group. In contrast, the T1DM group treated with AKK maintained a relatively stable body weight without a sharp decline, especially on day 14 (*p* = 0.0028). By the end of the treatment period, the group treated with AKK had a body weight similar to the control group, suggesting a protective effect of AKK against body weight loss in diabetic mice.

The impact of AKK and butyric acid on blood glucose levels in diabetic mice was assessed (Fig. 2D). Blood glucose levels showed a sharp increase in both untreated and treated T1DM groups on day 3, which persisted over time. Butyric acid treatment resulted in a moderate improvement of blood glucose levels in T1DM mice on day 18 compared to the untreated T1DM group (*p* = 0.033). Treatment with AKK notably decreased blood glucose levels on day 14 (*p* = 0.011). However, blood glucose levels subsequently increased again, similar to the butyric acid-treated group. Despite this fluctuation, AKK maintained a moderate effect on glucose levels.

During the study, AKK and butyric acid treatments affected illness severity in diabetic mice (Fig. 2E). The untreated group (T1DM) showed a continuous decline in symptoms over time. The butyric acid treatment helped reduce illness severity compared to the diabetic group, although its effects were moderate. The group treated with AKK showed a much more apparent improvement, with symptoms being significantly less severe than in the diabetic group (*p* = 0.005). By the end of the study, AKK had the most significant impact in reducing illness severity (*p* = 0.0003), showing a clear benefit over other treatments.

Food and water intakes were monitored throughout the study period (Fig. 2F and 2G). Diabetic symptoms were observed in diabetic groups with increased food and water intakes. However, by week 4, the AKK-treated group showed a moderate decrease in food consumption. The probiotic-treated group also showed reduced water intake compared to the untreated group during the experiment, notably at week 4.

### Effect of Probiotic and Butyric acid Treatment on Physiological Parameters of Diabetic Rats

**Fig. 3:**
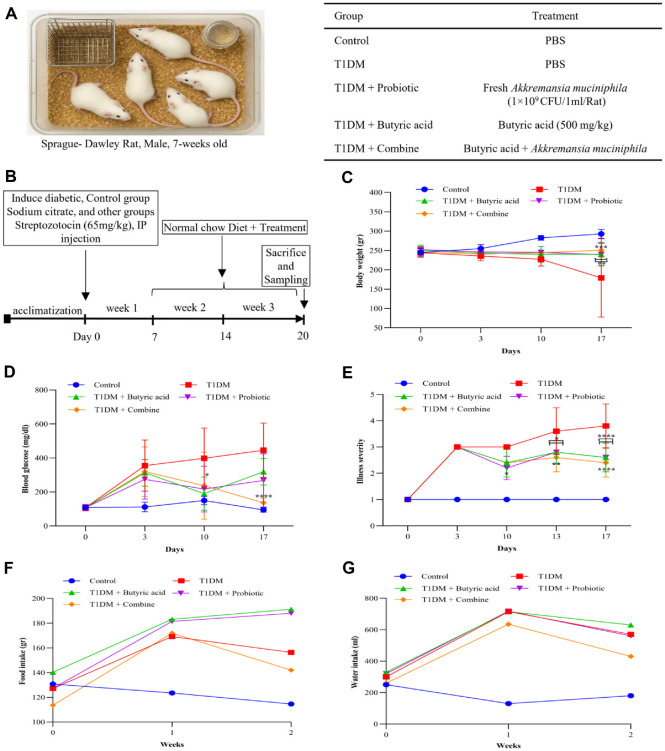
Therapeutic Effect of AKK and Butyric Acid in T1DM Rat Model. (**A**) Seven-week-old male SD rats were divided into the groups shown in the table. (**B**) After a week of acclimation period, rats in the T1DM and treatment groups received a single intraperitoneal injection of STZ (65 mg/kg) on day 0. Control rats received sodium citrate buffer. Treatments were administered daily from day 7 for 2 weeks, while the control group received PBS. (**C**) Body weights changed over the study period. Both monotherapy and combination treatment groups improved weight loss compared to the T1DM group, with the combination group showing the most substantial effect (***p < 0.001). (**D**) Blood glucose levels increased after STZ injection on day 0. After one week of treatment, all treatment groups showed reduced blood sugar levels compared to the T1DM group. By the second week, the combination therapy exhibited a marked improvement, with glucose levels approaching those of the control group(****p < 0.0001). AKK and butyric acid-treated groups also showed fluctuations, but to a lesser extent. (**E**) The illness severity scoring system (1 – healthy; 2 - minimally ill; 3 - moderately ill; 4 - severely ill; and 5 - dead) was applied across the groups. Treatments positively affected the rats' clinical condition compared to the T1DM group on day 13. The most significant reduction in severity was observed by the end of the study (****p < 0.0001). (**F, G**) Food and water intake were monitored. T1DM rats consumed less food and more water, while treatment groups showed partial recovery. The combination group displayed the most normalized intake patterns. Data are shown as mean ± SD for each group (*n* = 5). Statistical differences were evaluated relative to the T1DM group using two-way ANOVA followed by Tukey’s post hoc test (*p < 0.05; **p < 0.01; ***p < 0.001; ****p < 0.0001).

To investigate the therapeutic effects of AKK, butyric acid, and their combination on T1DM, male SD rats (7 weeks old) were used in five groups: control, T1DM, T1DM + butyric acid, T1DM + probiotic, and T1DM + combination (Fig. 3A). Diabetes was induced using a single STZ injection intraperitoneally at 65 mg/kg, followed by probiotic and butyric acid treatments. Sodium citrate buffer was injected into the control group. Diabetes was confirmed by blood glucose levels exceeding 250 mg/dL. Thereafter, daily oral treatments were started. The probiotic group received AKK at a concentration of 1 × 10^9^ CFU/1 ml. The butyric acid group received sodium butyrate at 500 mg/kg. The combination group received AKK at 1 × 10^9^ CFU/1 ml and butyric acid at 500 mg/kg (Fig. 3B).

Following the treatment, we evaluated the physiological parameters of diabetic rats. Induction of diabetes resulted in significant body weight loss across all groups. AKK and butyric acid treatments, especially when combined, attenuated diabetes-induced weight reduction (*p* = 0.0061 for AKK vs T1DM, *p* = 0.0052 for butyrate vs T1DM, and *p* = 0.0007 for combination therapy vs T1DM), indicating a preventive effect against diabetes-associated weight loss (Fig. 3C). As expected, blood glucose levels were significantly higher in diabetic rats. While both butyric acid and AKK treatments helped lower these levels, the combination therapy group showed the greatest of improvement, highlighting a potential synergistic effect in controlling blood sugar (*p* < 0.0001)(Fig. 3D). The severity of illness was monitored (Fig. 3E). Butyric acid and AKK treatment moderately reduced illness severity on day 13 (*p* = 0.015), while their combination demonstrated significant improvements (*p* = 0.001). By the end of the experiment, all treatment groups, especially the combination therapy, demonstrated a highly significant decline in disease severity (*p* < 0.0001).

Food and water intakes were monitored (Fig. 3F-3G). Both the butyric acid and AKK groups of rats consumed more food than the diabetic group, with water intakes showing similar levels to those of rats in the untreated group. However, the combination treatment group showed reductions in food and water intake. Data are presented as mean ± SD (*n* = 5 rats per group). Statistical significance was assessed using two-way ANOVA with Tukey’s post hoc test.

### Combination Therapy Promotes the Production of SCFAs in Diabetic Rats

**Fig. 4:**
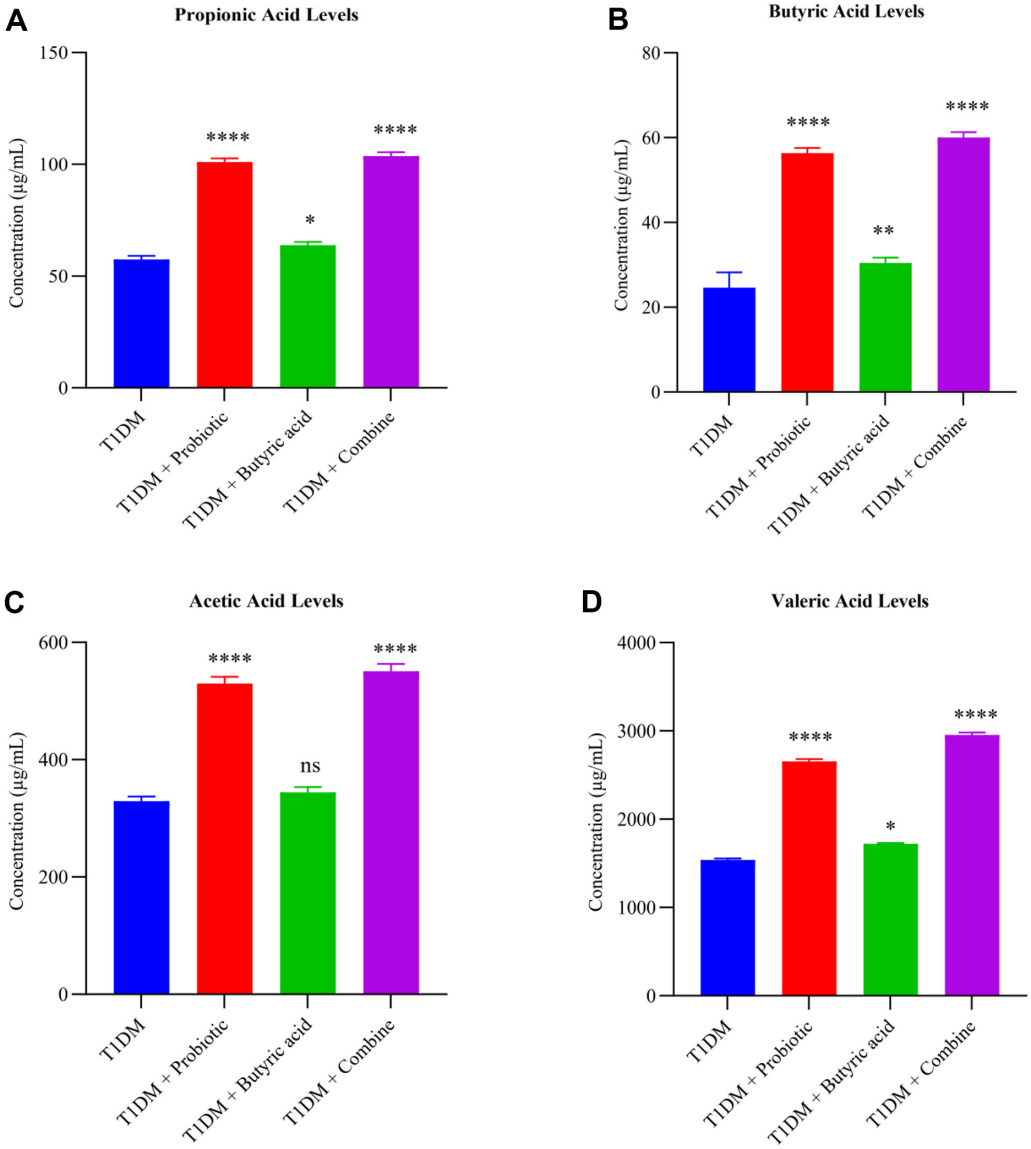
Detection of short-chain fatty acid concentrations in rat liver samples. (**A**) Propionic acid levels were increased more significantly in AKK and combination groups compared to the T1DM and butyric acid-treated groups. (**B**) A bar chart showing butyric acid levels. Its levels in probiotic and combined groups increased significantly compared to those in the butyric acid treatment group, but butyric acid also showed a significant increase. (**C**) A bar chart showing acetic acid levels. Acetic acid levels in all treatment groups increased compared to those in the T1DM group, with probiotic and combined treatment having better effects on the level of this acid. (**D**) A bar graph demonstrating valeric acid levels after different treatments. Similar to other SCFAs, a significant effect of the probiotics, especially the combined therapy, was observed. Data are presented as mean ± SD (*n* = 5 rats per group). Statistical significance was evaluated in comparison with the T1DM group using two-way ANOVA with Tukey’s post hoc test (*p < 0.05; **p < 0.01; ***p < 0.001; ****p < 0.0001).

SCFA concentrations were measured in rat livers at the end of the experiment (Fig. 4). Total levels of SCFAs were increased in rats treated with AKK and butyrate, and their combination compared to untreated T1DM rats (Fig. 4A). Probiotics and combination therapy significantly increased propionate levels (*p* < 0.001) compared to the diabetic group, while butyric acid alone produced a smaller increase (*p* < 0.05). Concentrations of butyric acid in probiotic-treated and combination therapy-treated groups were significantly increased (*p* < 0.0001) compared to the diabetic group, while butyrate alone only induced a moderate increase (*p* < 0.01) (Fig. 4B). Acetic acid level analysis showed a significant difference between treated and untreated T1DM rats. Acetic acid levels were significantly higher in the probiotic and combination therapy groups (*p* < 0.0001), whereas butyric acid alone had no effect (Fig. 4C). Similarly, valeric acid levels increased noticeably in the probiotic and combination groups compared to T1DM rats (*p* < 0.0001), while butyrate alone had a weaker effect (Fig. 4D).

These results show that supplementation with probiotics and butyric acid, especially their combination, restored SCFA levels in diabetic conditions, which may support metabolic regulation. Although an internal standard was not included in this analysis due to technical limitations, but relative comparisons between groups remain valid.

### Investigating the Protective Effects of Probiotics and Butyric Acid In Vitro on STZ-Induced Cytotoxicity

**Fig. 5:**
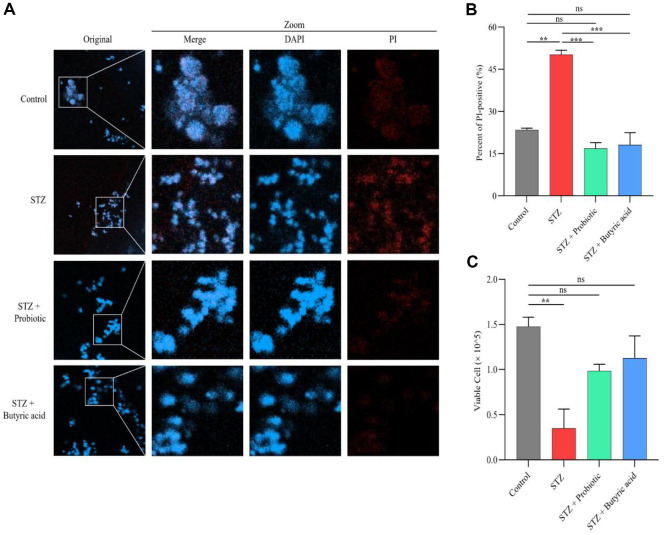
Protective effects of probiotics and butyric acid against STZ-induced cell damage. (**A**) PI fluorescence intensity was measured to assess cell death. STZ-treated cells exhibited a significant increase in PI fluorescence, indicating extensive cell damage. However, probiotic or butyric acid treatment reduced PI intensity, suggesting a protective effect against STZ-induced cytotoxicity. (**B**) Quantification of PI fluorescence confirmed that STZ exposure led to a significant increase in cell damage, which was markedly reduced by probiotic or butyric acid treatment, potentially supporting cell survival. (**C**) Cell viability analysis demonstrated that STZ treatment significantly decreased survival rates. However, probiotic or butyric acid supplementation significantly improved cell viability, highlighting their protective effects against STZ-induced toxicity. The statistical significance of the obtained data was determined compared to the untreated group using a one-way analysis of variance (**p < 0.01; ***p < 0.001).

We investigated the effects of probiotic (AKK) and butyric acid on STZ-induced cytotoxicity using 3T3-L1 cells by evaluating PI fluorescence intensity and cell viability (Fig. 5). PI fluorescence intensity, an indicator of cell death, was markedly increased in STZ-treated cells (50%), while treatment with probiotic or butyric acid reduced dead cells to about 15% and 20% respectively (Fig. 5A and 5B). Fig. 5C shows that cell viability decreased significantly in the STZ-treated group. Trypan blue assays showed a 2–3 fold increase in viable cells after the treatment. Together, this finding implies that AKK and butyric acid can promote cell survival or help repair damaged cells and reduce the adverse effects of STZ on cell viability, suggesting a protective effect of these treatments against STZ-induced cytotoxicity.

## Discussion

T1DM is an autoimmune disease where T lymphocytes mistakenly attack insulin-producing β-cells in the pancreas. Over time, this immune-mediated destruction reduces insulin production and makes it difficult for the body to regulate blood sugar levels [22]. In this study, our findings demonstrated that both AKK and butyrate improved metabolic parameters, with combination therapy showing synergistic effects on body weight, glycemic control, illness severity, and SCFA production. Furthermore, *in vitro* studies revealed that these treatments protected against STZ-induced cytotoxicity in 3T3-L1 cells.

The growth pattern of AKK in our study exhibited that AKK robustly grows under strict anaerobic conditions with peak levels observed around 42 h in the anaerobic chamber, which best simulates the colon’s oxygen-free environment. This time point corresponds to the log phase of bacterial growth, which is typically associated with maximum metabolic activity and production of bioactive compounds. We collected samples during this phase to maximize the therapeutic potential of the bacterial metabolites used in downstream applications [23].

Animal models, especially rodents, are generally used to induce diabetes mellitus because they can help us understand the complex causes of the disease and how it affects the entire body, which *in vitro* methods alone cannot fully show [24].

We first established an optimized model of diabetes in C57BL/6 mice using STZ. This optimization allowed us to evaluate the effects of AKK and butyrate treatments in a subsequent experiment [14]. According to our findings, a single injection of STZ at 150 mg/kg effectively induced hyperglycemia, as evidenced by increased blood glucose levels, moderate body weight loss, and increased illness severity without causing excessive mortality. Our study aligns with other STZ studies, indicating that 150 mg/kg STZ offers a good balance of efficacy and tolerability in diabetic mouse models [14].

After establishing the diabetic model, diabetic mice were treated with AKK and butyric acid once daily for 2 weeks. AKK improved overall health and reduced diabetic symptoms more effectively than butyric acid treatment alone. While we did not directly assess the underlying mechanisms, the observed improvements in overall health and reduction of diabetic symptoms with AKK treatment might be supported by previous studies. These studies demonstrate that AKK could decrease gut permeability, lowering inflammation and protecting pancreatic islets from immune attacks [25, 26]. Similarly, previous studies support butyrate’s ability to reduce blood glucose levels while having little effect on body weight [27].

We extended the experiment to a rat model to further evaluate our findings. Rats provide a closer metabolic and physiological relationship to humans; therefore, they are valuable models for researching human diseases and biology [28]. The STZ dose of 65 mg/kg was used for T1DM induction in rats based on previous studies that aimed to balance effective diabetes induction with low mortality and high model reproducibility [15, 29]. Prior studies on STZ-induced rat models show that the 65 mg/kg dose achieves substantial β-cell destruction (about 84% reduction in pancreatic insulin content within 24 h), leading to long-lasting hyperglycemia and a stable T1DM phenotype without excessive mortality [30]. We administered AKK, butyrate, and their combination in a rat model. The results demonstrated that combined therapy resulted in noticeable reductions in physical parameters and illness severity. Although both AKK and butyric acid individually showed beneficial effects, their combination led to greater improvements in blood glucose control, weight maintenance, and symptom reduction. This suggests a possible synergistic effect, where the combined treatments may enhance each other's actions and lead to better overall outcomes than when used alone.

Mice and rats in the STZ group consumed more water and food. This is because excessive urination due to high blood sugar levels caused by diabetes leads to increased thirst and food intake.

Diabetes and liver health are closely interconnected, with diabetes-related hepatic complications such as NAFLD (non-alcoholic fatty liver disease) also associated with disturbances in hepatic metabolism and insulin signaling [31, 32]. Thus, in light of this association, we tested SCFA levels in liver tissue to explore potential metabolic changes associated with our treatments, given their role as key metabolic factors and immune function. The hepatic acetate, propionate, and butyrate levels were especially elevated in the probiotics/butyric acid combination-treated rats. Our results relate to other research suggesting that gut microbiota and their metabolites, such as SCFAs, are involved in the regulation of host energy balance and immune response [33]. The mucus layer of the gut was reported to be enriched with producers of SCFA through AKK, and SCFAs absorbed in the colon reach the liver via portal circulation, thus may have an impact on hepatic metabolism [34, 35].

Our SCFA analysis in liver tissue showed that the levels of acetate, propionate, butyrate, and valeric acid were significantly increased in the probiotic and butyrate treatment groups compared to the diabetic control. SCFA levels were highest following the combination therapy, consistent with a synergistic effect or complementary effect between AKK and butyric acid. While we did not evaluate gut-derived compounds in this study, previous studies have indicated that increased levels of SCFAs may be indicative of improved microbial metabolism and host-microbe interactions in conditions where these processes are disturbed, such as diabetes [36, 37]. However, additional research is required to validate this interaction.

To understand the cellular mechanisms of the therapeutic effects of AKK and butyric acid, we conducted *in vitro* experiments using 3T3-L1 cells exposed to STZ. Our findings demonstrated that both treatments significantly improved cell viability and decreased PI fluorescence, suggesting protective effects against STZ-induced cytotoxicity. These results align with previous studies showing that STZ treatment significantly increases cytotoxicity and inhibits insulin secretion, as evidenced by an increased number of PI-positive cells, which is indicative higher level of apoptosis or necrosis under diabetic conditions [21]. However, AKK and butyrate treatment can increase insulin levels and decrease cell death [21]. Collectively, these results suggest that both therapies may help maintain cellular integrity and function under diabetic conditions.

## Conclusion

In summary, our study suggests that Akk alone and in combination with sodium butyrate can play an important role in treating diabetes in murine models. They can also regulate the disturbance of SCFA metabolites in the liver. Although these findings are promising, more research is necessary to understand their mechanisms and how they can be used effectively to manage diabetes.

## Supplemental Materials

Supplementary data for this paper are available on-line only at http://jmb.or.kr.
