# Supplementary material for: In Vivo Therapeutic Potential of Next-Generation Probiotic Akkermansia muciniphila and Butyrate Combination Therapy in Diabetes
Source: J Microbiol Biotechnol. 2025 Oct 28;35:e2506025. doi: 10.4014/jmb.2506.06025 (PMC12603377; doi:10.4014/jmb.2506.06025)
Supplement: Supplementary file 1 [file jmb-35-e2506025-supple.pdf]

J. Microbiol. Biotechnol. 2025. 35: e2506025

https://doi.org/10.4014/jmb.2506.06025

Supplementary Figure

Figure S1

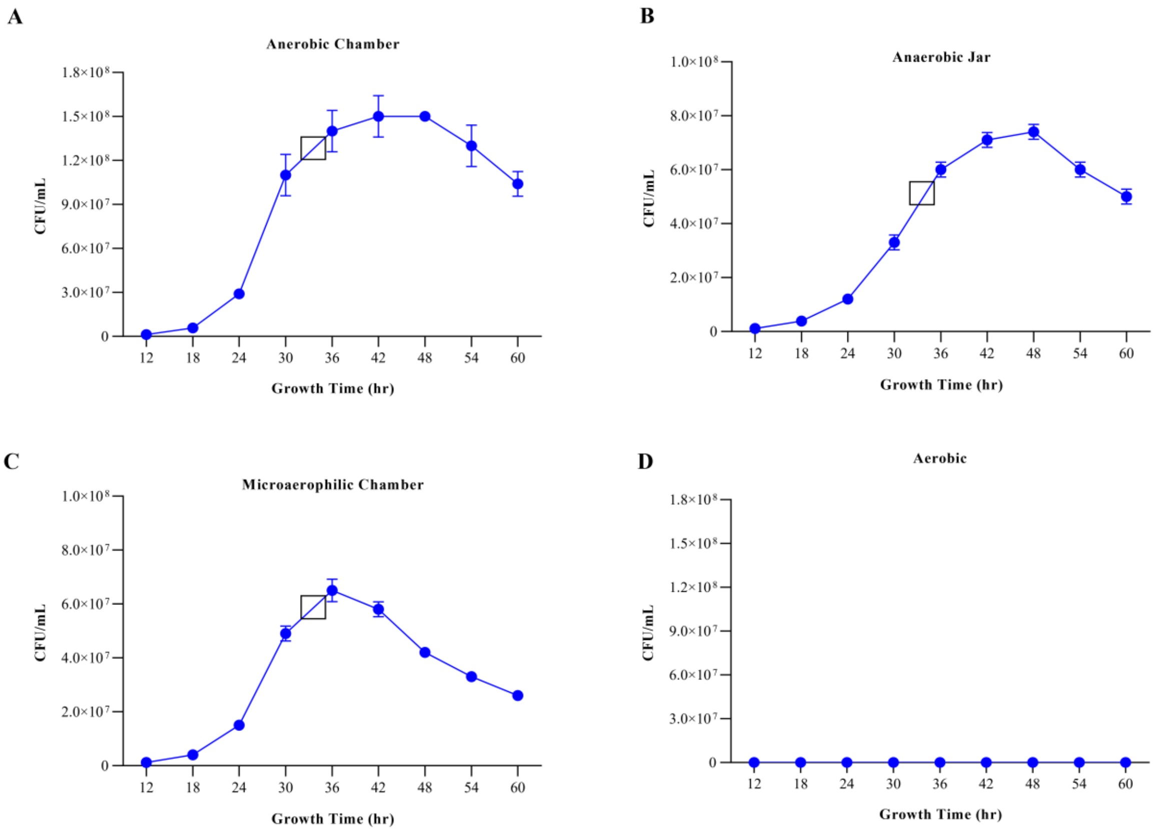

Fig. S1. Growth curves of AKK under different atmospheric conditions.

Growth was assessed every 6 h by CFU/mL enumeration under four cultivation environments: **(A)** Anaerobic Chamber, **(B)** Anaerobic Jar with anaerobic catalyst, **(C)** Microaerophilic Chamber, and **(D)** Aerobic Condition. The strain showed optimal growth in the anaerobic chamber, peaking at ~ 1.5×10^8 CFU/mL by 42 h. Moderate growth (~ 7.6×10^7 CFU/mL) was recorded in the anaerobic jar, while limited proliferation was observed in the microaerophilic chamber, with a peak of ~ 6.8×10^7 CFU/mL. No detectable growth occurred under aerobic conditions, confirming the strict anaerobic nature of the strain. The rectangular box in each plot denotes the log (exponential) phase of growth, evident between 30–36 h in anaerobic and partially anaerobic conditions. Data represent means of biological duplicates; error bars show the range.
